# Screening Tea Cultivars for Novel Climates: Plant Growth and Leaf Quality of *Camellia sinensis* Cultivars Grown in Mississippi, United States

**DOI:** 10.3389/fpls.2020.00280

**Published:** 2020-03-13

**Authors:** Qianwen Zhang, Tongyin Li, Qiushuang Wang, Judson LeCompte, Richard L. Harkess, Guihong Bi

**Affiliations:** ^1^Department of Plant and Soil Sciences, College of Agriculture and Life Sciences, Mississippi State University, Mississippi State, MS, United States; ^2^Tea Research Institute, Guangdong Academy of Agricultural Sciences, Guangdong Key Laboratory of Tea Plant Resources Innovation and Utilization, Guangzhou, China

**Keywords:** tea, *Camellia sinensis*, plant growth, cold tolerance, leaf quality, polyphenols, amino acids, caffeine

## Abstract

The United States (U.S.) consumed over 80 billion servings of tea, approximately 3.8 billion gallons, in the year of 2018. With the vast majority of tea demand being met by importation, the United States became the third largest tea importer worldwide after Russia and Pakistan. As demand for domestically produced tea increases and growers expressing increasing interest in growing and producing tea, tea production became an emerging industry in the United States. Compared to major tea producing countries with centuries of growing history, tea production in the United States is limited and requires research support in many aspect of tea production including selecting suitable cultivars adapted to local climatic conditions. This study evaluated nine tea cultivars, including ‘BL1,’ ‘BL2,’ ‘Black Sea,’ ‘Christine’s Choice,’ ‘Dave’s Fave,’ ‘Large Leaf,’ ‘Small Leaf,’ ‘Sochi,’ and ‘var. *assamica*,’ for plant growth, leaf morphological characteristics, cold tolerance, and leaf biochemical compositions when grown in Mississippi United States with a subtropical climate. The nine tested cultivars had varying plant growth indices (PGI) and varying degrees of cold tolerance to freezing temperatures in winter, but resumed healthy growth the following spring. ‘BL2’ showed the highest PGI of 104.53 cm by February 2019, which might be helpful toward suppressing weed and early establishment of tea plantation. The nine cultivars also showed varying leaf characteristics in terms of leaf length, width, area, fresh and dry weights, and new shoot weight. There existed a diversity in leaf biochemical composition including soluble solids, carbohydrates, total polyphenols (TP), free amino acids (AA), L-theanine and caffeine among the nine cultivars and among different harvesting seasons of spring, summer, and fall within a certain cultivar. The nine cultivars in this study generally grow well in local environment. All tea samples collected from nine cultivars and three seasons were considered suitable for green tea processing with low TP/AA ratios ranging from 1.72 to 3.71 in this study.

## Introduction

Tea is the most popular beverage worldwide second only to water, with world consumption of 5.5 million tons in 2016 (Food and Agriculture Organization of the United Nations [FAO], 2018). The industry value for world tea consumption increased from $1.84 billion in 1990 to $12.66 billion in 2018, with a projected strong increasing trend over the next 10 years (Goggi, 2018; USDA, 2018). Tea is rich in a number of health beneficial compounds including catechin, caffeine, theanine, and other polyphenols. Polyphenols in tea are antioxidants believed to slow down aging, prevent certain types of cancer, and reduce risk of cardiovascular diseases (Sharangi, 2009; Lorenzo and Munekata, 2016). Americans consumed over 3.8 billion gallons, or more than 84 billion servings of tea in 2018, making the United States the third largest importer of tea after Russia and Pakistan, importing a total of 263 million pounds including black and green tea (Tea Association of the U.S.A., 2019). The vast majority of tea demand in the United States is met by importation. With increased demand for locally sourced food products, questions are raised regarding whether domestic production of tea is feasible.

Tea plant is a broad-leaved evergreen shrub, adapted to subtropical to tropical climates, with optimal growing temperatures of 18 to 30°C during the growing season and the ability to withstand temperatures from −16 to 40°C (Bhagat et al., 2010; Luo, 2015). They thrive in warm, humid climates with annual rainfall of 1,250 to 6,000 mm, favor humidity levels of 80 to 90% and elevations up to 2,000 m above sea level (Hajra, 2001). Successful tea plant production requires deep, light, well-drained, and acid soil with pH ranging from 4.5 to 5.5 (Willson and Clifford, 1992; Hajra, 2001; Ruan et al., 2007; Gascoyne et al., 2016). In the United States tea can be grown in USDA hardiness zones 6 to 9 (Dirr, 2009). Once established, a tea plantation can have commercial productivity for decades (Wang, 2016).

As United States growers seek to diversify their crops, tea plants can potentially serve as a high-value alternative crop. Tea production effort occurs in over 16 states in the United States, including Alabama, California, Florida, Georgia, Hawai’i, Idaho, Louisiana, Maryland, Michigan, Mississippi, New Jersey, New York, North Carolina, Oregon, South Carolina, Texas, Virginia, and Washington with the majority started over the past decade and having limited production (Zee et al., 2003; Walcott, 2012; Song et al., 2012; Bell, 2014; Hardin, 2017; LeCompte, 2018). The state of Mississippi is located in a subtropical climate, having annual average temperatures ranging from 17°C in the north to 20°C along the coast. Annual precipitation ranges from 1270 to 1650 mm (50–65 inches) and fairly evenly distribute throughout the year (Mississippi State University, 2019). There are currently three small scale commercial tea farms in Mississippi. Growing tea in Mississippi is subjected to challenges including periods of drought and flood, temperatures exceeding 35°C for over 100 days annually, and potential cold damage with lowest temperatures down to −14°C. Compared to centuries of growing history of tea in leading tea producing countries including China and India (Food and Agriculture Organization of the United Nations [FAO], 2015), there lacks research-based information to guide farmers on growing tea in the United States. The availability of suitable cultivars adapted to local climates is fundamental to an emerging tea industry in the United States.

Tea germplasms have been evaluated using morphology, biochemical compositions, molecular markers, and sensory evaluations (Feng et al., 2014; Li Y. et al., 2016; Wambulwa et al., 2016). Fresh leaf characteristics including leaf size, area, and fresh weight are commonly evaluated in breeding programs for yield potential. The final quality of tea product is highly associated with leaf biochemical composition including polyphenols, soluble solids, carbohydrates, amino acids (AA), theanine and caffeine (Willson and Clifford, 1992; Li X. et al., 2016). There exists large variations in physical characteristics and chemical composition in leaves of different germplasms (Willson and Clifford, 1992; Gai et al., 2019). Growing environment including soil, temperature, precipitation, light, seasonality, and altitude affect leaf characteristics and biosynthesis of important chemicals, thus the final tea quality (Lee et al., 2010; Wang et al., 2011; Wei et al., 2011; Han et al., 2017; Ahmed et al., 2019). This study is of the few recent research efforts to evaluate tea cultivars grown in Southeastern United States. The objectives of this study were to investigate plant growth and leaf morphology of nine tea cultivars; and to investigate chemical compositions in leaves of these cultivars in different seasons of the year (spring, summer, and fall) when grown in Mississippi, United States.

## Materials and Methods

### Plant Cultivation

One-year-old tea plants propagated from stem cuttings grown in one-gallon containers were transplanted into the field located at the R. R. Foil Plant Research Center at Mississippi State University (USDA Hardiness Zone 8a; 33°29′N 88°47′W) in spring 2017. The nine tested cultivars included: ‘BL1,’ ‘BL2,’ ‘Black Sea,’ ‘Christine’s Choice,’ ‘Dave’s Fave,’ ‘Large leaf,’ ‘Small leaf,’ ‘Sochi,’ and ‘var. *assamica*.’. Tea plants were pruned to a uniform height of 30.48 cm (12 inch) at transplanting and grown in full sun in Stough fine sandy loam soil with a pH of 4.9 (Brent, 1973). Plants were planted in a double row hedge, with 0.76 m (2.5 ft) between plants within a row, 0.91 m (3 ft) between rows within the double row, and 1.83 m (6 ft) apart between double rows center-to-center. Each plant was fertilized with controlled release fertilizer 15N-3.9P-10K (Osmocote^®^ Plus, 15-9-12, 8–9 months, ICL Specialty Fertilizers, Summerville, SC, United States) at a rate of 110 g per plant per year based on recommended medium rate. All plants were irrigated as needed through drip irrigation. Wheat straw was used between rows to aid weed control.

### Plant Growth and Cold Tolerance

Each plant was measured for plant height, width 1 and width 2, where width 1 was the greatest width of an individual plant and width 2 was the perpendicular width to width 1, in February 2018 and 2019. Plant Growth Index (PGI) was calculated as the average of plant height, width 1, and width 2. Plants were evaluated for cold tolerance in February 2018 and 2019. The percentage of foliage showing cold-damaged symptoms on each plant was recorded as described by Luo (2015). All plants were pruned to a height of 30.48 cm (12 inch) in 2018 and 50.80 cm (20 inch) in 2019 after plant growth and cold tolerance data were collected in February. Local monthly air temperature data, including average, maximum, and minimum temperatures, within the experiment duration were obtained from the USDA Natural Resources Conservation Service website (U. S. Department of Agriculture, National Resources Conservation Service [USDA-NRCS], 2019).

### Leaf Characteristics

Leaf characteristics of each cultivar including individual leaf length, width, area, fresh, and dry weights were evaluated in Feb. 2018. For each cultivar, twenty most recent fully expanded leaves were collected from each replication (block) composed of 20 plants, with a total of four replications. Each leaf was measured for length and width (widest points apart). The twenty leaves from each replication were then passed through a leaf area meter (LI-3100C; LI-COR Biosciences, Lincoln, NE, United States) for the total leaf area, and an average was calculated for individual leaf area. Fresh weight of the 20 leaves from each replication was measured, and an average individual leaf fresh weight was calculated. The leaves were then oven dried at 60°C until no weight change for their average dry weight. Fresh weight of 100 new shoots in each cultivar, composed of one terminal bud and two leaves, were also measured with four replications.

### Photosynthetic Activities

Plant photosynthetic activity was measured between 1,000 and 1,300 _HR_ on September 20, 2018 using a portable photosynthesis system (LI-6400XT; LI-COR Biosciences, Lincoln, NE, United States). One plant from each replication was randomly selected to measure photosynthetic activities. For each plant, one most recent fully expanded leaf, not shaded by other leaves, was enclosed into a 2 cm^2^ leaf chamber for the photosynthetic measurements. Photosynthetically active radiation (*PAR*) of 1500 μmol m^–2^ s^–1^ and reference CO_2_ concentration of 400 μmol mol^–1^ were maintained inside the leaf chamber during measurements. Block temperature in the leaf chamber was maintained according to the air temperature on the measurement date. Net photosynthetic rate (*P*_n_), stomatal conductance (*g*_*s*_), and leaf transpiration rate (Trmmol) were measured on each selected plant.

### Preparation of Tea Extract

New shoots containing one terminal bud and two leaves were harvested on 10 April, 12 July, and 18 October 2018 to represent tea harvests in spring (first flush of growth), summer, and fall. New shoots from each harvest were then oven-dried at 60°C and ground to pass a 40-mesh (0.42 mm) sieve using a Wiley mini mill (Thomas Scientific, Waltham, MA, United States). Dry shoot sample of 0.6 g were infused with 100 mL freshly boiled deionized water for 45 min. Then the supernatant was filtered through filter paper (Grade 1, GE Healthcare Bio-Sciences Corp., Marlborough, MA, United States) using a vacuum pump. After filtration, deionized water was added to the supernatant to reach a final volume of 100 mL. Three tea extracts (subsamples) were prepared for each replication and then used to test for biochemical compositions including soluble solids, carbohydrates, total polyphenols, free AAs, L-theanine and caffeine content. All biochemical compositions were presented as percentage on a dry weight basis.

### Soluble Solids

Soluble solid content was measured following the protocol of Xu et al. (2018) with minor modifications. Tea extract of 50 mL was added to a weighed evaporation dish and evaporated to dryness, then oven-dried at 120°C (for approximately 2 h) to a constant weight and then cooled to room temperature in a desiccator. The residual solids were then measured to calculate soluble solid content.

### Carbohydrates

Carbohydrates in tea extract were measured by modified anthrone-sulphuric acid method using dextrose as the standard as described by Fan et al. (2017). Anthrone-sulphuric acid solution (1 g L^–1^ anthrone dissolved in sulphuric acid) of 8 mL was added to 1 mL of tea extract. Then the mixture was placed in a water bath at 100°C for 7 min. After cooling to room temperature, absorbance of the solution at 620 nm was determined using a 1 cm photometer disposable cuvette and a spectrophotometer (Nicolet evolution 100, Thermo Scientific, Waltham, MA, United States). Dextrose, anthrone and sulfuric acid reagent were equal or above to ACS grade, purchased from Thermo Fisher Scientific (Waltham, MA, United States).

### Caffeine

Caffeine content in tea extract was analyzed by high performance liquid chromatography (HPLC) (1260 Infinity II series; Agilent Technologies, Willington, DE, United States) according to Wang et al. (2019) with modifications. Tea extract was filtered through a 0.22 μm membrane. HPLC analyses were performed using a diode array detector (G1315C Diode-array Detector, Agilent Technologies) with an injection volume of 10 μL, flow rate of 1 mL min^–1^, controlled oven temperature of 30°C, and a C18 column [Agilent TC-C18 (2), 4.6 mm × 250 mm, 5 μm; Agilent Technologies] plus a C18 guard column [Agilent TC-C18 (2) Grd, 4.6 mm × 12.5 mm; Agilent Technologies]. Mobile phase A consists of 0.5% acetic acid, 1% acetonitrile, and 2% methanol. Mobile phase B consists of 0.5% acetic acid, 10% acetonitrile, and 20% methanol. The elution program was 0–30 min: percentage of mobile phase A linearly decreased from 72.5 to 20.0%, percentage of mobile phase B linearly increased from 27.5 to 80.0%; 30–35 min: percentage of mobile phase A linearly increased from 20.0 to 72.5%, percentage of phase B linearly decreased from 80.0 to 27.5%; 35–40 min: 72.5% mobile phase A and 27.5% mobile phase B. Chromatograms were recorded at 280 nm. Caffeine used to develop calibration standard curves were purchased from Sigma-Aldrich (St. Louis, MO, United States). Mobile phase of HPLC grade were purchased from Thermo Fisher Scientific (Waltham, MA, United States).

### Total Polyphenols

Total polyphenols in tea extract were estimated using the Folin-Ciocalteu method described by Singleton and Rossi (1965). The tea extract was diluted 25 times with deionized water. Then 2.5 mL of freshly prepared 10% (v/v) Folin & Ciocalteu’s phenol reagent (Sigma-Aldrich Co., St. Louis, MO, United States) was added to 0.5 mL diluted tea extract. After 5 min of equilibration, 2 mL of 75 g L^–1^ Na_2_CO_3_ was added to the mixture, which was then placed at room temperature for 60 min. The absorbance of the solution at 765 nm was measured in a glass cuvette using a spectrophotometer. Folin-Ciocalteu reagent was purchased from Sigma-Aldrich Company. The calibration standard curve was obtained using gallic acid (Sigma-Aldrich Co.) as standard.

### Free Amino Acids

The content of free AAs was determined by the ninhydrin dyeing method as described by Yin et al. (2009) with minor modifications using glutamic acid as standard. Ninhydrin solution (40 g L^–1^) of 0.5 mL was added to 1.0 mL of tea extract in a test tube with cap. Then 0.5 mL of pH 8.0 buffer (95% v/v 0.067 mol L^–1^ Na_2_HPO_4_⋅12H_2_O solution and 5% v/v 0.067 mol L^–1^ KH_2_PO_4_ solution) was added to the mixture. Test tubes with caps were then placed in a water bath at 100°C for 15 min. After cooling to room temperature, the solution was transferred to a volumetric flask and diluted to 25 mL with deionized water. Absorbance of the diluted solution at 540 nm was determined using a 1 cm photometer disposable cuvette and a spectrophotometer. Glutamic acid, ninhydrin, Na_2_HPO_4_⋅12H_2_O, KH_2_PO_4_ were equal or above to ACS grade, purchased from Thermo Fisher Scientific.

### L-Theanine

L-theanine in tea extract was determined by HPLC as described by Li et al. (2019) with minor modification using L-theanine as standard. HPLC analyses were performed using the same detector and columns as mentioned in caffeine analyses. The injection volume was 20 μL of 0.22 μm membrane filtered tea extract, flow rate was 2 mL min^–1^, controlled oven temperature was set at 27°C. Mobile phase A was 100% water and mobile phase B was 100% acetonitrile. The elution program was 0–7 min: 100% A; 7–9 min: percentage of mobile phase B linearly increased from 0 to 60%; 9–15 min: percentage of mobile phase B linearly decreased from 60 to 0%; 15–20 min: 100% A. Chromatograms were recorded at 210 nm. L-theanine used to develop calibration standard curves were purchased from Sigma-Aldrich (St. Louis, MO, United States).

### Principal Components Analyses (PCA)

A PCA was conducted to investigate the relationship among nine tested cultivars using six biochemical descriptors including contents of soluble solids, carbohydrates, caffeine, total polyphenol, free AAs, L-theanine and the TP/AA ratios. Tea leaf samples used in the PCA were collected from each cultivar and from three harvesting seasons including spring, summer, and fall of 2018 as mentioned above. The PCA was conducted using software XLSTAT (version 2019; Addinsoft United States, New York, NY, United States).

### Experimental Design and Data Analyses

The experiment was conducted in a completely randomized block design with four replications and cultivar as the experimental factor. There were 20 single-plant subsamples for each cultivar within each replication. The number of subsamples used for each variable varied and was specified in each methodology section. Significance of the cultivar effect was determined by the analysis of variance (ANOVA) using the PROC GLM procedure of SAS (version 9.4; SAS Institute, Cary, NC, United States). A logarithmic transformation was made on cold damage data (%) to meet the assumption of normality. Means were separated by Tukey’s honestly significant difference (HSD) test at *P* ≤ 0.05. All statistical analyses were performed using SAS.

## Results

### Local Air Temperatures

**FIGURE 1:**
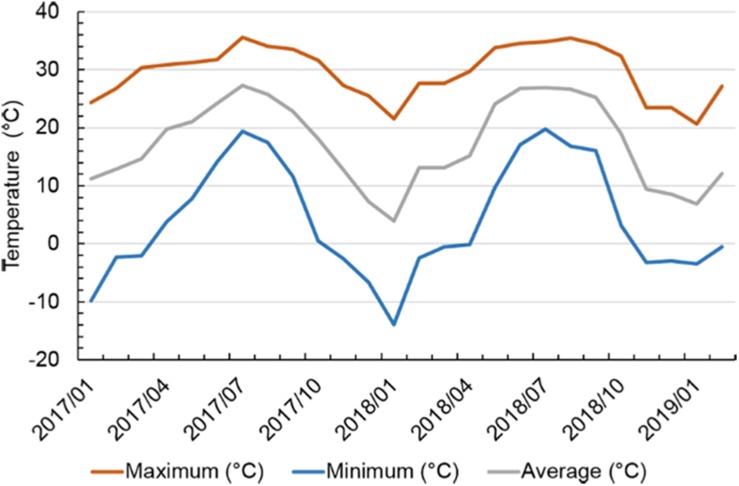
Maximum, minimum, and average air temperatures on a monthly basis in Starkville, MS, United States from January 2017 to February 2019. Air temperature data were obtained from the website of USDA Natural Resources Conservation Service (https://wcc.sc.egov.usda.gov/reportGenerator/).

Within the experiment duration from January 2017 to February 2019, average monthly air temperature ranged from 3.9°C in January 2018 to 27.3°C in July 2017 (Figure 1 and Supplementary Figure S1). Maximum monthly air temperature ranged from 20.6°C in January 2019 to 35.6°C in July 2017. Minimum monthly air temperature ranged from −13.9°C in January 2018 to 19.8°C in July 2018. The lowest air temperature in this study was −13.9°C, and occurred in January 2018 in which the winter was colder than normal in this region. The highest air temperature was 35.6°C and occurred in July 2017.

### Plant Growth Indices

Tea cultivars varied in size with plant growth indices ranging from 38.22 to 59.34 cm in February 2018 (Table 1, Supplementary Figure S1). ‘BL2’ had the highest PGI of 59.34 cm. The second highest PGIs were in ‘Black Sea,’ ‘Christine’s Choice,’ and ‘Large Leaf’ ranging from 50.44 to 53.36 cm. ‘BL1,’ ‘Small Leaf,’ and ‘var. *assamica*’ had the lowest PGIs ranging from 38.22 to 41.25 cm. In February 2019, the trend of PGI among cultivars was similar to 2018, ranging from 63.75 to 104.53 cm. ‘BL2’ had the highest PGI of 104.5 cm, and ‘BL1,’ ‘Small Leaf,’ and ‘var. *assamica*’ had the lowest PGIs of 69.33, 63.75, and 74.62 cm, respectively. ‘Black Sea,’ ‘Christine’s Choice,’ ‘Dave’s Fave,’ and ‘Large Leaf’ had comparable PGIs, but lower than ‘BL2,’ ranging from 83.97 to 92.92 cm. From February 2018 to February 2019, PGIs of all cultivars increased by 62.0–83.6%.

**TABLE 1:** Plant growth index (PGI) of nine tea cultivars grown in Mississippi, United States.

	**Plant growth index (cm)**	
**Cultivar**	**February 2018**	**February 2019**
BL1	38.22 ± 1.19e	69.33 ± 3.19ef
BL2	59.34 ± 1.08a	104.53 ± 1.22a
Black Sea	53.36 ± 0.9b	88.97 ± 1.46b
Christine’s Choice	50.44 ± 0.71b	83.97 ± 4.18bc
Dave’s Fave	46.19 ± 0.65c	84.79 ± 1.09bc
Large Leaf	51.92 ± 0.76b	92.92 ± 0.99b
Small Leaf	39.35 ± 0.84e	63.75 ± 1.46f
Sochi	45.00 ± 0.86cd	79.81 ± 1.77cd
var. *assamica*	41.25 ± 1.29de	74.62 ± 3.42de
*P*-value	<0.0001	<0.0001

**One-year-old tea plants were transplanted into the field in spring 2017. Plant growth index was calculated as the average of plant height, width 1 (widest points apart), and width 2 (perpendicular to width 1) and measured in February 2018 and February 2019. Different lower-case letters within a column suggest significant difference among cultivars indicated by Tukey’s HSD test at P ≤ 0.05*.*

### Cold Tolerance

Measured in February 2018, ‘Christine’s Choice’ showed the highest percentage of cold damage, with an average of 34.67% foliage on an individual plant showing symptoms (Table 2). ‘Dave’s Fave’ showed the second highest percentage of cold damage of 18.2% foliage per plant. ‘BL2,’ ‘Small Leaf,’ and ‘Sochi’ had the least cold damage with average cold-damaged foliage of 2.08–3.85%. In February 2019, cold damage on tea plants was not as severe compared to February 2018 due to a generally mild winter. ‘Dave’s Fave’ and ‘Small Leaf’ had the highest percentage of cold-damaged foliage ranging from 9.83 to 9.90%. In comparison, ‘BL2’ and ‘Large Leaf’ showed the least cold-damaged foliage at 1.90 and 1.94%, respectively.

**TABLE 2:** Cold damage of nine tea cultivars in February 2018 and February 2019 grown in Mississippi, United States.

	**Cold damage (% foliage per plant)**	
**Cultivar**	**February 2018**	**February 2019**
BL1	6.11 ± 1.34def	5.64 ± 0.66bc
BL2	3.25 ± 0.36fg	1.94 ± 0.25d
Black Sea	7.51 ± 1.24cde	6.73 ± 0.54ab
Christine’s Choice	34.67 ± 2.08a	4.79 ± 0.57c
Dave’s Fave	18.15 ± 2.18b	9.83 ± 1.63a
Large Leaf	7.32 ± 1.13c	1.90 ± 0.18d
Small Leaf	3.85 ± 0.55efg	9.90 ± 1.10a
Sochi	2.08 ± 0.23g	5.03 ± 0.67c
var. *assamica*	13.71 ± 3.21cd	7.40 ± 1.28bc
*P*-value	< 0.0001	<0.0001

**Cold damage on each plant was evaluated as the percentage of foliage showing cold-damaged symptoms and became brown in color. Different lower case letters within a column suggest significant difference among cultivars indicated by Tukey’s HSD test at P < 0.05.**

### Leaf and Shoot Characteristics

The nine tested cultivars had varying leaf shapes and sizes (Table 3). ‘Large Leaf’ had the largest leaf length of 9.61 cm per leaf. Lower than ‘Large Leaf,’ ‘BL2,’ ‘Black Sea,’ ‘Christine’s Choice,’ ‘Dave’s Fave,’ ‘Sochi,’ and ‘var. *assamica*’ had similar leaf lengths ranging from 8.04 to 8.73 cm per leaf. ‘BL1’ and ‘Small Leaf’ had the lowest leaf lengths averaging less than 8 cm per leaf. In terms of leaf width, ‘Dave’s Fave’ and ‘Large Leaf’ had the widest leaves and were wider than the other seven cultivars. The smallest leaf width was in ‘BL1’ averaging 2.99 cm per leaf. The other six cultivars had leaf widths ranging from 3.40 cm in ‘Small Leaf’ to 4.12 cm in ‘Christine’s Choice.’

**TABLE 3:** Leaf and shoot characteristics of nine tea cultivar grown in Mississippi, United States.

**Cultivar**	**Leaf length (cm)**	**Leaf width (cm)**	**Leaf area (cm^2^)**	**Fresh leaf weight (g)**	**Dry leaf weight (g)**	**New shoot weight (g per 100 shoot)**
BL1	7.52 ± 0.22c	2.99 ± 0.06e	13.11 ± 0.85e	0.50 ± 0.06e	0.22 ± 0.03e	35.50 ± 1.91ef
BL2	8.60 ± 0.13b	3.60 ± 0.07cd	20.74 ± 0.97bcd	0.80 ± 0.06cd	0.37 ± 0.03cd	38.48 ± 1.78de
Black Sea	8.04 ± 0.17bc	3.57 ± 0.06cd	19.66 ± 1.62cd	0.80 ± 0.08cd	0.37 ± 0.04cd	29.23 ± 1.36f
Christine’s Choice	8.65 ± 0.15b	4.12 ± 0.07b	23.88 ± 1.43bc	0.99 ± 0.08bc	0.44 ± 0.03bc	48.26 ± 2.21c
Dave’s Fave	8.18 ± 0.08bc	4.76 ± 0.06a	26.07 ± 0.83ab	1.17 ± 0.06ab	0.52 ± 0.02ab	63.40 ± 2.98b
Large Leaf	9.61 ± 0.10a	4.80 ± 0.06a	29.43 ± 0.69a	1.40 ± 0.04a	0.58 ± 0.02a	74.60 ± 2.86a
Small Leaf	7.56 ± 0.18c	3.40 ± 0.08d	16.62 ± 0.60de	0.61 ± 0.04de	0.28 ± 0.02de	28.85 ± 1.67f
Sochi	8.73 ± 0.16b	3.72 ± 0.07c	22.92 ± 1.35bc	0.92 ± 0.02bc	0.42 ± 0.01bc	39.95 ± 1.17de
var. *assamica*	8.06 ± 0.17bc	3.61 ± 0.10cd	20.85 ± 1.34bcd	0.84 ± 0.08cd	0.38 ± 0.04cd	43.73 ± 1.18cd
*P*-value	<0.0001	<0.0001	<0.0001	<0.0001	<0.0001	<0.0001

**Different lower case letters within a column suggest significant difference among cultivars indicated by Tukey’s HSD test at P < 0.05*.*

Single leaf areas of the nine cultivars ranged from 13.11 cm^2^ in ‘BL1’ to 29.43 cm^2^ per leaf in ‘Large Leaf’ (Table 3). ‘BL2,’ ‘Christine’s Choice,’ ‘Dave’s Fave,’ ‘Sochi,’ and ‘var. *assamica*’ had comparable single leaf areas ranging from 19.66 to 26.07 cm^2^ per leaf. ‘BL1’ had the smallest single leaf area, lower than any of the other eight cultivars.

Individual leaf fresh or dry weight shared a similar trend with leaf area (Table 3). ‘Large Leaf’ and ‘Dave’s Fave’ had the greatest leaf weight, fresh (1.40 g and 1.17 g per leaf, respectively) or dry (0.58 g and 0.52 g per leaf, respectively). The five cultivars ‘BL2,’ ‘Black Sea,’ ‘Christine’s Choice,’ ‘Sochi,’ and ‘var. *assamica*’ had comparable leaf fresh weights ranging from 0.80 to 0.99 g per leaf, and dry weights ranging from 0.37 to 0.44 g per leaf. ‘BL1’ and ‘Small Leaf’ had the lowest leaf fresh weights (0.50 g and 0.61 g per leaf, respectively) and dry weights (0.22 g and 0.28 g per leaf, respectively).

Fresh weight of 100 new shoots of the nine cultivars ranged from 28.85 g in ‘Small Leaf’ to 74.60 g in ‘Large Leaf’ (Table 3). ‘Dave’s Fave,’ ‘Christine’s Choice’ had intermediate new shoot weight of 63.4 and 48.26 g per 100 shoots, respectively. ‘BL1,’ ‘Black Sea,’ and ‘Small Leaf’ had the comparable lowest fresh weight of 100 new shoots.

### Photosynthetic Activity Measurements

Tea cultivars did not vary significantly in net photosynthetic rate (Pn), stomatal conductance (*g*_s_), or leaf transpiration rate (Trmmol) when measured in September 2018 (Table 4). Pn ranged from 5.53 μmol m^–2^ s^–1^ in ‘var. *assamica*’ to 11.51 μmol m^–2^ s^–1^ in ‘BL2.’ Stomatal conductance and leaf transpiration rate ranged from 0.10 to 0.19 mol m^–2^ s^–1^ and 3.19 to 5.69 mmol m^–2^ s^–1^, respectively, suggesting cultivars were at similar physiological and water status.

**TABLE 4:** Net photosynthetic rate, stomatal conductance, and leaf transpiration rate of nine tea cultivar grown in Mississippi, United States.

**Cultivar**	***P*_n_ (μmol m^–2^ s^–1^)**	***g*_s_ (mol m^–2^ s^–1^)**	**Trmmol (mmol m^–2^ s^–1^)**
BL1	9.81 ± 0.55	0.19 ± 0.03	5.69 ± 0.05
BL2	11.51 ± 1.08	0.14 ± 0.04	4.35 ± 0.66
Black Sea	6.68 ± 1.14	0.14 ± 0.04	4.07 ± 0.74
Christine’s Choice	6.93 ± 0.90	0.14 ± 0.02	4.19 ± 0.21
Dave’s Fave	8.03 ± 2.69	0.15 ± 0.03	4.66 ± 0.54
Large Leaf	10.86 ± 1.64	0.17 ± 0.03	5.03 ± 0.15
Small Leaf	7.98 ± 0.99	0.15 ± 0.05	4.29 ± 0.85
Sochi	9.11 ± 2.89	0.17 ± 0.07	4.62 ± 1.18
var. *assamica*	5.53 ± 1.59	0.1 ± 0.04	3.19 ± 0.98
*P*-value	0.26	0.90	0.53

**Pn, net photosynthetic rate; g_*s*_, stomatal conductance; Trmmol, leaf transpiration rate. P > 0.05 indicates no significant difference among cultivars within a column by Tukey’s HSD test*.*

### Soluble Solids

**FIGURE 2:**
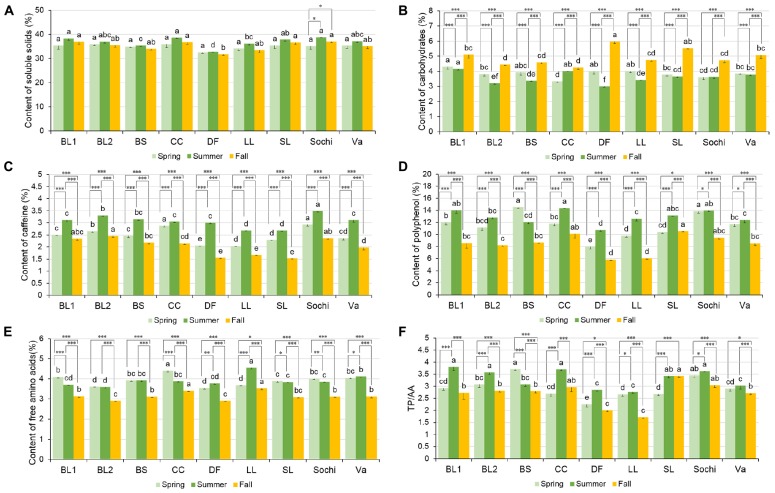
Content of soluble solids (dry weight%) **(A)**, carbohydrates (dry weight%) **(B)**, caffeine (dry weight%) **(C)**, total polyphenols (dry weight%) **(D)**, free amino acids (AA) (dry weight%) **(E)**, and ratio between total polyphenols and free amino acids (TP/AA) **(F)** in tea extract of nine cultivars grown in Mississippi, United States. ‘BS’ stands for cultivar ‘Black Sea,’ ‘CC’ stands for cultivar ‘Christine’s Choice,’ ‘DF’ stands for cultivar ‘Dave’s Fave,’ ‘LL’ stands for cultivar ‘Large Leaf,’ ‘SL’ stands for cultivar ‘Small Leaf,’ ‘Va’ stands for cultivar ‘var. *assamica*.’ Different lower case letters on top of the same colored bars suggest significant difference in soluble solid content among nine cultivars within one harvesting season indicated by Tukey’s HSD test at *P* ≤ 0.05; ^∗^ suggests significant difference within one cultivar among different harvest seasons at *P* ≤ 0.05; ^∗∗^ suggests significant difference within one cultivar among different harvest seasons at *P* ≤ 0.01; ^∗∗∗^ suggests significance different within one cultivar among different harvest seasons at *P* ≤ 0.0001.

Among nine cultivars, soluble solids in tea extract ranged from 32.6 to 36.02%, 32.89 to 38.68%, and 31.87 to 37.15% on a dry weight basis in spring, summer, and fall of 2018, respectively (Figure 2A). In spring, there was no difference in soluble solid content among the nine cultivars. In summer, six cultivars including ‘BL1,’ ‘BL2,’ ‘Christine’s Choice,’ ‘Small Leaf,’ ‘Sochi,’ and ‘var. *assamica*’ had comparable soluble solids content ranging from 36.97 to 38.84%. ‘Dave’s Fave’ had the lowest soluble solids content of 32.89%. In fall, nine cultivars generally had similar soluble solids with ‘Dave’s Fave’ having the lowest soluble solids of 31.87%, lower than ‘BL1,’ ‘Christine’s Choice,’ ‘Small Leaf,’ and ‘Sochi.’ There was generally no difference in soluble solids content among seasons within a certain cultivar, except for ‘Sochi’ having higher soluble solids content in summer and fall than in spring.

### Carbohydrates

Carbohydrates in nine cultivars ranged from 3.33 to 4.31%, 3.00 to 4.16%, and 4.24 to 5.99% in spring, summer, and fall, respectively (Figure 2B). In spring, ‘BL1,’ ‘Black Sea,’ ‘Dave’s Fave,’ and ‘Large Leaf’ had comparable highest carbohydrates ranging from 3.94 to 4.31%, with’ Christine’s Choice’ having the lowest carbohydrate content of 3.33%. In summer, ‘BL1’ and ‘Christine’s Choice’ had the highest carbohydrates at 4.16 and 4.02% respectively, higher than ‘BL2,’ ‘Black Sea,’ ‘Dave’s Fave,’ ‘Large Leaf,’ ‘Small Leaf,’ or ‘Sochi.’ In fall, ‘Dave’s Fave’ and ‘Small Leaf’ had the highest carbohydrates at 5.99 and 5.53%, respectively. ‘BL2,’ ‘Black Sea,’ ‘Christine’s Choice,’ ‘Large Leaf,’ and ‘Sochi’ had comparable low carbohydrate content ranging from 4.24 to 4.76%. Within each cultivar, carbohydrates in fall were the highest among the three tested seasons. In seven cultivars including ‘BL1,’ ‘BL2,’ ‘Black Sea,’ ‘Dave’s Fave,’ ‘Large Leaf,’ ‘Small Leaf,’ and ‘var. *assamica*,’ spring carbohydrates were higher than summer, whereas in ‘Christine’s Choice’ higher carbohydrates was found in summer than in spring.

### Caffeine

Caffeine content in nine cultivars ranged from 2.06 to 2.92%, 2.67 to 3.49%, and 1.53 to 2.46% in spring, summer, and fall, respectively (Figure 2C). The trend of caffeine levels among nine cultivars was generally similar in all three seasons. ‘Sochi’ had the highest levels of caffeine among cultivars in spring and summer. ‘Dave’s Fave,’ ‘Large Leaf,’ and ‘Small Leaf’ generally had the lowest caffeine levels in all three seasons. All nine cultivars shared the same trend of caffeine levels across seasons, where cultivars had the highest caffeine content in summer, spring the second, with fall having the lowest caffeine content.

### Total Polyphenols (TP)

Total polyphenols in nine cultivars ranged from 7.99 to 14.53%, 10.75 to 14.36%, and 5.83 to 10.55% in spring, summer, and fall, respectively (Figure 2D). In spring, ‘Black Sea’ and ‘Sochi’ had the highest total polyphenol content of 14.53 and 13.82%, respectively. Lower than ‘Black Sea’ or ‘Sochi,’ ‘BL1,’ ‘BL2,’ ‘Christine’s Choice,’ and ‘var. *assamica*’ had comparable total polyphenol ranging from 11.72 to 11.98%. ‘Dave’s Fave’ had the lowest total polyphenol of 7.98%. In summer, ‘BL1,’ ‘Chiristina’s Choice,’ ‘Small Leaf,’ and ‘Sochi’ had comparable highest total polyphenol content ranging from 13.15 to 14.36%, whereas ‘Dave’s Fave’ had the lowest total polyphenol of 10.75%. In fall, the highest polyphenol content was found in ‘Christine’s Choice’ (10.11%), ‘Small Leaf’ (10.55%), and ‘Sochi’ (9.48%), and the lowest polyphenol was found in ‘Dave’s Fave’ (5.83%) and ‘Large Leaf’ (6.05%). All cultivars except ‘Black Sea’ shared a similar trend in total polyphenols across seasons: highest total polyphenol was found in summer and lowest in fall. ‘Black Sea’ had the highest polyphenol of 14.53% in spring, higher than summer of 12.03%, with fall being the lowest of 8.69%.

### Free Amino Acids (AA)

The content of free AAs in nine cultivars ranged from 3.54 to 4.41%, 3.59 to 4.56%, and 2.91 to 3.54% in spring, summer, and fall, respectively (Figure 2E). In spring, ‘Christine’s Choice’ had the highest total free AAs at 4.41%, with ‘BL2,’ ‘Dave’s Fave,’ and ‘Large Leaf’ having the lowest total AAs ranging from 3.54 to 3.67%. In summer, ‘Large Leaf’ had the highest free AAs at 4.56%. In fall, ‘Christine’s Choice’ and ‘Large Leaf’ had the highest total free AAs of 3.41 and 3.54%, respectively. Five cultivars, ‘BL1,’ ‘Black Sea,’ ‘Small Leaf,’ ‘Sochi,’ and ‘var. *assamica*,’ had comparable free AAs ranging from 3.09 to 3.14%. Cultivars varied in trends of free AAs among the three seasons. In ‘BL1,’ ‘Christine’s Choice,’ ‘Small Leaf,’ and ‘Sochi,’ greatest free AAs were found in spring, summer second, with fall having the lowest AAs. In ‘BL2’ and ‘Black Sea,’ spring and summer had comparable free AAs, both higher than fall. In ‘Dave’s Fave,’ ‘Large Leaf,’ and ‘var. *assamica*’ greatest free AAs were found in summer, spring the second, with fall being the lowest.

### L-Theanine

The content of L-theanine in nine cultivars ranged from 0.28 to 0.87%, 0.33 to 0.99%, and 0.47 to 0.85% in spring, summer and fall, respectively (Table 5). In spring, ‘Christine’s Choice’ had the highest L-theanine content of 0.87%, and ‘Small Leaf’ had the second highest L-theanine content of 0.68%, with ‘Dave’s Fave’ having the lowest content of 0.28%. Six cultivars including ‘BL1,’ ‘BL2,’ ‘Black Sea,’ ‘Large Leaf,’ and ‘Small Leaf’ had comparable intermediate L-theanine content ranging from 0.36 to 0.47%. In summer, ‘Large Leaf’ had the highest L-theanine content of 0.99%, with ‘BL1,’ ‘BL2,’ ‘Dave’s Fave,’ and ‘Sochi’ having comparable lowest content of 0.33 to 0.41%. In fall, six cultivars including ‘Black Sea,’ ‘Christine’s Choice,’ ‘Large Leaf,’ ‘Small Leaf,’ ‘Sochi,’ and ‘var. *assamica*’ had comparable L-theanine content of 0.67 to 0.85%, with ‘BL1’ having the lowest content of 0.47%. Among seasons, L-theanine content was generally the highest in the fall among all cultivars. ‘BL2,’ ‘Black Sea,’ ‘Sochi,’ and ‘var. *assamica*’ had comparable L-theanine contents in spring and summer. ‘BL1,’ ‘Christine’s Choice,’ and ‘Small Leaf’ had higher L-theanine content in spring than in summer, with ‘Dave’s Fave’ and ‘Large Leaf’ having higher content in summer than spring.

**TABLE 5:** Content of L-theanine in tea extract in three harvest seasons and nine cultivars grown in Mississippi, United States.

	L-Theanine(%)		
Cultivar	Spring	Summer	Fall
BL1	0.47 ± 0.04 Acd	0.33 ± 0.02 Bd	0.47 ± 0.05 Ad
BL2	0.36 ± 0.03 Bde	0.33 ± 0.02 Bd	0.59 ± 0.03 Abcd
Black Sea	0.43 ± 0.04 Bd	0.51 ± 0.03 Bc	0.74 ± 0.07 Aab
Christine’s Choice	0.87 ± 0.10 Aa	0.53 ± 0.04 Bbc	0.85 ± 0.05 Aa
Dave’s Fave	0.28 ± 0.02 Ce	0.36 ± 0.03 Bd	0.53 ± 0.06 Acd
Large Leaf	0.42 ± 0.03 Bd	0.99 ± 0.09 Aa	0.84 ± 0.06 Aa
Small Leaf	0.68 ± 0.04 Ab	0.53 ± 0.03 Bbc	0.67 ± 0.05 Aabc
Sochi	0.44 ± 0.03 Bd	0.41 ± 0.02 Bcd	0.76 ± 0.05 Aab
var. *assamica*	0.56 ± 0.04 Bc	0.64 ± 0.04 Bb	0.77 ± 0.04 Aab
*P*-value	<0.0001	<0.0001	<0.0001

**Different lower case letters within a column suggest significant difference in L-theanine content among nine cultivars within one harvesting season indicated by Tukey’s HSD test at P < 0.05. Different capitalized letters within a row suggest significant different in L-theanine content among three seasons within a certain cultivar indicated by Tukey’s HSD test at P < 0.05*.*

### TP to AA Ratio

The ratio between total polyphenol and free amino acids (TP/AA) among nine cultivars ranged from 2.26 to 3.71, 2.77 to 3.80, and 1.72 to 3.42 in spring, summer, and fall, respectively (Figure 2F). In spring, the highest ratios of TP/AA were found in ‘Black Sea’ and ‘Sochi,’ with ‘Dave’s Fave’ having the lowest TP/AA ratio of 2.26. In summer, the five cultivars ‘BL1,’ ‘BL2,’ ‘Christine’s Choice,’ ‘Small Leaf,’ and ‘Sochi’ had comparable high ratios of TP/AA ranging from 3.43 to 3.80. In fall, the highest ratios of TP/AA were found in ‘Christine’s Choice,’ ‘Small Leaf,’ and ‘Sochi,’ with ‘Dave’s Fave’ and ‘Large Leaf’ having the lowest ratios. Six cultivars ‘BL1,’ ‘BL2,’ ‘Christina’s Choice,’ ‘Dave’s Fave,’ ‘Large Leaf,’ and ‘Sochi’ had higher ratios of TP/AA ratios in summer than in spring or fall. ‘Black Sea’ and ‘var. *assamica*’ had higher TP/AA ratios in spring and summer than in fall.

### Principle Component Analyses (PCA)

**FIGURE 3:**
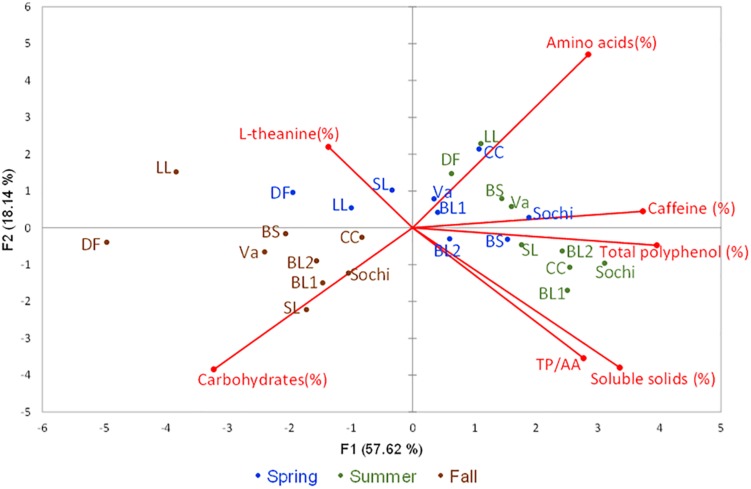
Principle component analyses (PCA) in nine tea cultivars grown in Mississippi, United States with six descriptors. The principal components scatterplot was generated based seven descriptors including contents of soluble solids, carbohydrates, total polyphenols, caffeine, L-theanine, and the ratios between total polyphenols and free amino acids (TP/AA) using leaf samples collected from nine test tea and from three harvesting seasons from each cultivar in spring (blue color), summer (green color), and fall (brown color) of 2018. The first and second principal components accounted for 75.75% of the total variation. ‘BS’ stands for cultivar ‘Black Sea,’ ‘CC’ stands for cultivar ‘Christine’s Choice,’ ‘DF’ stands for cultivar ‘Dave’s Fave,’ ‘LL’ stands for cultivar ‘Large Leaf,’ ‘SL’ stands for cultivar ‘Small Leaf,’ ‘Va’ stands for cultivar ‘var. *assamica*.’

The first and second principle components (PC1 and PC2) in PCA accounted for 57.62 and 18.14% of the variation among the nine cultivars tested in three seasons, respectively (Figure 3). The positive PC1 dimension was largely correlated with contents of soluble solids, caffeine, total polyphenols, AAs, and TP/AA that had higher values in spring or summer. PC1 was negatively correlated with content of carbohydrates and L-theanine that had higher values in fall. The positive PC2 dimension was largely correlated with contents of L-theanine and AAs. PC2 was negatively correlated with content of carbohydrates, soluble solids and TP/AA. Based on the PCA results, tea leaves of nine cultivars harvested from fall were generally characterized with high content of carbohydrates. Except for ‘Dave’s Fave’ and ‘Large Leaf,’ tea leaves collected from spring which were characterized with a high content of AAs. Except for ‘Black Sea,’ leaves of the other cultivars collected in summer were characterized with high contents of total polyphenols and caffeine.

## Discussion

The nine tested cultivars in this study, including ‘BL1,’ ‘BL2,’ ‘Black Sea,’ ‘Christine’s Choice,’ ‘Dave’s Fave,’ ‘Large leaf,’ ‘Small leaf,’ ‘Sochi,’ and ‘var. *assamica’* showed diverse results in plant growth, leaf morphology and chemical composition. Within each cultivar, leaf chemical composition also varied among harvesting seasons in spring, summer, and fall. Despite some cold damage during winter, the nine tested cultivars generally demonstrated healthy growth and satisfactory adaptation to the growing environment in Mississippi since spring 2017.

At this current stage of tea industry in the United States, availability of superior tea cultivars are of the most limiting factors. To our knowledge, ‘BL1,’ ‘BL2,’ ‘Black Sea,’ ‘Christine’s Choice,’ ‘Dave’s Fave,’ ‘Large leaf,’ ‘Small leaf,’ and ‘Sochi’ are *Camellia sinensis* var. *sinensis*. Var. *assamica* is *Camellia sinensis* var. *assamica*. As for sources of tested cultivars, two cultivars ‘BL1’ and ‘BL2’ were donated by Robert E. “Buddy” Lee from Transcend Nursery in Louisiana, they were grown in the nursery since mid-1970s and survived down to −13°C during the 1980s. The remaining seven cultivars were obtained from Camellia Forest Nursery in North Carolina. Through personal communication with the nursery, ‘Black Sea’ was collected from Batumi region of Georgia and grown in the nursery since 2015; ‘Christine’s Choice’ and ‘Dave’s Fave’ were collected from China and grown in the nursery since 2008; ‘Sochi’ was collected from Sochi region of Russia and grown in the nursery since 2008; ‘Large Leaf,’ ‘Small Leaf,’ and ‘var. *assamica*’ were grown in the nursery since 1970s, 1970s, and 2000 with unknown original sources, respectively. The exact genotype of tea cultivars remain unclear. Research is in progress to investigate genetic background of tested cultivars using single nucleotide polymorphisms (Yamashita et al., 2019), and will be discussed in future research.

Tea is a long-lived woody perennial plant with decades of commercial productivity (Wang, 2016). Previous studies in traditional tea producing countries focused on profitable tea plants that were over 4 years and up to 100 years in age (Han et al., 2007; Kamau et al., 2008; Chan et al., 2009; Kumar et al., 2015). Few studies have been conducted in newly transplanted tea fields. The tea plants in our field were pruned back to 30 cm in February 2018 (2 years old) and to 50 cm in February 2019 (3 years old), respectively. The increase in PGI of tea plants in our study ranged from 62.0 to 83.6% within a year, which is consistent with previous studies (Green, 1971; Kulasegaram and Kathiravetpillai, 1972; Ahmed et al., 2014). When tea plants have a high growth rate and tea canopy covers 90% of tea fields, weed control becomes less of a concern (Wang, 2016). Fast plant growth and increases in PGI are thus beneficial for newly established tea fields to suppress weed growth (Anderson, 2010). Larger plant sizes, measured by PGI, may serve as an indicator for yield, which is one of the most important consideration in commercial tea production. Therefore, tea plants with higher PGI may establish faster, decrease weed pressure, and potentially become profitable at a younger age. In our study, ‘BL2’ had the highest PGI, followed by ‘Black Sea,’ ‘Christine’s Choice,’ and ‘Large Leaf.’

Leaf morphology, including size and weight, are correlated with tea yield (Karthigeyan et al., 2008). Of the nine tested cultivars, ‘Large Leaf’ had the largest leaf length (9.61 cm), width (4.80 cm), area (29.43 cm^2^), and fresh (1.40 g) and dry weights (0.58 g) on a single leaf basis and the highest 100 new shoot weight of 74.60 g. Rajkumar et al. (2010) reported *C. sinensis* var. *assamica* to be a large leaf variety with leaf length of 13.05 cm and width of 5.35 cm when grown in India. In our study, leaf size of var. *assamica* was among the smallest of the nine tested cultivars, which may have been affected by genetic background, plant age, and growing environment (Parkhurst and Loucks, 1972; Forrester et al., 2017). ‘BL1’ had the smallest leaf length of 7.52 cm and width of 2.99 cm, appearing to be larger than some small leaf cultivars reported from China (Karthigeyan et al., 2008). Leaf morphology also affects suitability of tea cultivars to make certain types of tea. For instance, most black tea is made from large leaf varieties (Astill et al., 2001). As plants age, leaf morphology of the tested cultivars may change.

Within this study duration, from spring 2017 to spring 2019, the lowest air temperature was reported to be −13.9°C in January 2018 (U. S. Department of Agriculture, National Resources Conservation Service [USDA-NRCS], 2019), which was approximately the historical extreme in this region. Tea plants can tolerate lowest air temperatures between −6 and −16°C, varying among varieties and different growth environments (Bi et al., 2014). With approximate 1 week of minimum air temperature below −6°C in January 2018, ‘Christine’s Choice’ had the most severe cold damage in February 2018, with over 34% of foliage showing cold damage symptoms. However, PGIs of ‘Christine’s Choice’ increased by 66% in spring 2019, suggesting the cultivar resumed vigorous growth after relatively severe cold damage. It was reported that cold damage on shoots may serve as a pinching agent, reducing apical dominance, as seen in *Cordyline*, and may promote production of more lateral shoots (Harris et al., 2001). In addition to the pinching effect of cold damage, all plants were pruned to a fixed height (30.48 and 50.80 cm in 2018 and 2019, respectively). Removal of apical dominance also promote lateral growth during the growing season. Compared to 2018, minimum air temperature in Jan 2019 was −3.5°C, resulting in cold damage on all cultivars below 10%. Lu et al. (2017) reported that tea cultivars varied in their critical cold temperature below which cell damage increased rapidly in tea leaves. This might explain the more severe cold damages in January 2018 compared to January 2019.

A wide range of variation in leaf chemical composition existed among the nine tested cultivars and among the three harvest seasons. This is in agreement with previous reports, where a high variability in soluble solids, total polyphenols, carbohydrates, AAs and caffeine content was found due to differences in gene expression and environmental conditions among cultivars and harvest seasons (Xu W. et al., 2012; Jayasekera et al., 2014; Fang et al., 2017). The content of soluble solids affects sensory characteristics of tea extract, including cream formation, viscosity, mouth feel and taste (Xu Y. Q. et al., 2012; Wang et al., 2019). Eight tested cultivars had similar soluble solids contents among the three harvest seasons, except that ‘Sochi’ had the highest soluble solids content in summer and lowest in spring. In comparison, the nine tested cultivars showed more variations among three seasons in their contents of carbohydrates, total polyphenols, free AAs, and caffeine.

With a number of reports around the world confirming seasonal effects on content of total polyphenol and AAs (Hilton et al., 1973; Gulati and Ravindranath, 1996; Yao et al., 2005; Chen et al., 2010; Lee et al., 2010; Jayasekera et al., 2014), few studies investigated the seasonal effects on carbohydrate content in tea leaves. In this study, the PCA results clearly separated fall samples from spring or summer due to the higher carbohydrate content in each cultivar (Figure 3). Such results indicate seasonality significantly affects carbohydrate content in tea leaves. A previous study reported the accumulation of carbohydrates contributes to increased cold tolerance of tea plants (Yue et al., 2015), suggesting that increasing carbohydrate levels in the fall might be related to plants adaptation to decreasing temperatures.

There are three fundamental tastes in tea extract including umami, sweetness, and bitterness. AAs, especially are an important contributor to the umami taste, which is especially important for green tea quality (Narukawa et al., 2008). The TP/AA ratio is a widely-used parameter to evaluate suitability of tea leaves to make certain types of tea. A previous study reported increased TP/AA with increasing temperatures (Han et al., 2017). Most of our results agreed with this finding except for ‘Black Sea’. The TP/AA ratios found in the nine tested cultivars in this study, ranging from 1.72 to 3.80 with total polyphenol content ranging from 5.83 to 14.53%, are considered suitable for green tea processing as black tea requires higher TP/AA ratios (Karori et al., 2007; Ai et al., 2011; Han et al., 2017). The low TP/AA ratios may have resulted from the young age of tea plants used in this study, having limited ability for biosynthesis of phenolics (Wang, 2013).

L-theanine is an important AAs in tea for its health benefits in reducing blood pressure, stress and anxiety, and improving memory and learning ability. L-theanine also contributes to flavor of tea infustion including caramel and umami tastes and alleviate bitterness (Yokogoshi et al., 1995; Juneja et al., 1999; Narukawa et al., 2008; Yang et al., 2013). Compared to the lowest AAs content in fall being among all seasons, L-theanine content was generally the highest in fall. This was consistent with Wang’s (2013) in investigating seasonal effects on tea leaves grown in China. While green tea harvested from spring were considered most popular with optimum quality in terms of flavor and taste (Liu et al., 2015), tea harvested from fall may contain higher health beneficial compounds considering the highest contents of total polyphenols and L-theanine among three seasons in tested cultivars. Future research will be conducted to investigate changes of individual AA agent among season and over time as tea plants mature.

## Conclusion

The nine cultivars tested in this study varied in plant size, leaf characteristics (including leaf length, width, area, fresh and dry weights, and new shoot weight), and leaf biochemical compounds including carbohydrates, TP, AA, caffeine, and L-theanine. ‘BL2’ showed the highest PGI of 104.53 cm by February 2019, a beneficial characteristic toward weed suppression and early establishment of tea plantation. ‘Large Leaf’ and ‘Dave’s Fave’ had the largest leaves in terms of individual leaf area, fresh, dry weights, and new shoot weight. Biochemical compounds in tea cultivars differed among harvesting seasons of spring, summer, and fall. With TP/AA ratios ranging from 1.72 to 3.71, the tested nine cultivars are generally considered suitable for green tea processing in three seasons at current growth stage, which may change over time as plants mature and requires further investigation.

## Data Availability Statement

All datasets generated for this study are included in the article/Supplementary Material.

## Author Contributions

GB, QZ, and JL designed and set up experiments. QZ and QW carried out experiments. QZ and TL analyzed experimental results. GB, RH, and TL provided essential instruments and technical guidance. TL and QZ wrote the manuscript that was revised and approved by GB, RH, QW, and JL.

## Disclaimer

Mention of a trademark, proprietary product, or vendor does not constitute a guarantee or warranty of the product by the United States Department of Agriculture and does not imply its approval to the exclusion of other products or vendors that also may be suitable.

## Conflict of Interest

The authors declare that the research was conducted in the absence of any commercial or financial relationships that could be construed as a potential conflict of interest.
