# Supplementary figures and images for: Screening Tea Cultivars for Novel Climates: Plant Growth and Leaf Quality of Camellia sinensis Cultivars Grown in Mississippi, United States
Source: Front Plant Sci. 2020 Mar 13;11:280. doi: 10.3389/fpls.2020.00280 (PMC7083152; doi:10.3389/fpls.2020.00280)

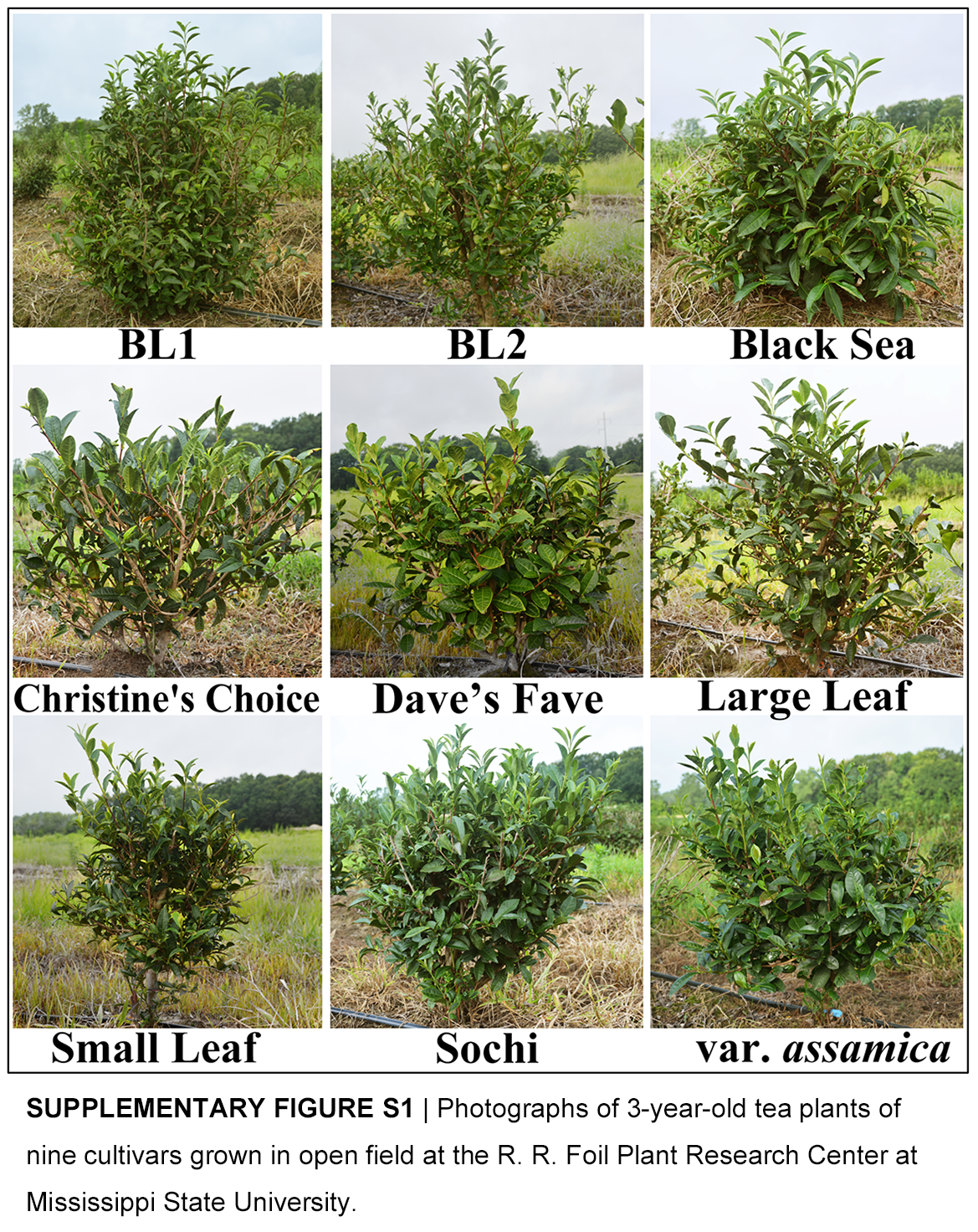

Supplement: Supplementary file 1 [file Image_1.tif]
